# Refining Our Understanding of the Flow Through Coronary Artery Branches; Revisiting Murray’s Law in Human Epicardial Coronary Arteries

**DOI:** 10.3389/fphys.2022.871912

**Published:** 2022-04-04

**Authors:** Daniel J. Taylor, Jeroen Feher, Ian Halliday, D. Rodney Hose, Rebecca Gosling, Louise Aubiniere-Robb, Marcel van ‘t Veer, Danielle Keulards, Pim A. L. Tonino, Michel Rochette, Julian Gunn, Paul D. Morris

**Affiliations:** ^1^ Department of Infection, Immunity and Cardiovascular Disease, University of Sheffield, Sheffield, United Kingdom; ^2^ ANSYS France, Villeurbanne, France; ^3^ Insigneo Institute for In Silico Medicine, Sheffield, United Kingdom; ^4^ Department of Cardiology, Sheffield Teaching Hospitals NHS Foundation Trust, Sheffield, United Kingdom; ^5^ Department of Cardiology, Catharina Hospital, Eindhoven, Netherlands; ^6^ Department of Biomedical Engineering, Eindhoven University of Technology, Eindhoven, Netherlands

**Keywords:** bifurcation, left main coronary artery, stable angina, translational physiology, Murray’s exponent

## Abstract

**Background:** Quantification of coronary blood flow is used to evaluate coronary artery disease, but our understanding of flow through branched systems is poor. Murray’s law defines coronary morphometric scaling, the relationship between flow (Q) and vessel diameter (D) and is the basis for minimum lumen area targets when intervening on bifurcation lesions. Murray’s original law (Q *α* D^P^) dictates that the exponent (P) is 3.0, whilst constant blood velocity throughout the system would suggest an exponent of 2.0. In human coronary arteries, the value of Murray’s exponent remains unknown.

**Aim:** To establish the exponent in Murray’s power law relationship that best reproduces coronary blood flows (Q) and microvascular resistances (Rmicro) in a bifurcating coronary tree.

**Methods and Results:** We screened 48 cases, and were able to evaluate inlet Q and Rmicro in 27 branched coronary arteries, taken from 20 patients, using a novel computational fluid dynamics (CFD) model which reconstructs 3D coronary anatomy from angiography and uses pressure-wire measurements to compute Q and Rmicro distribution in the main- and side-branches. Outputs were validated against invasive measurements using a Rayflow™ catheter. A Murray’s power law exponent of 2.15 produced the strongest correlation and closest agreement with inlet Q (zero bias, r = 0.47, *p* = 0.006) and an exponent of 2.38 produced the strongest correlation and closest agreement with Rmicro (zero bias, r = 0.66, *p* = 0.0001).

**Conclusions:** The optimal power law exponents for Q and Rmicro were not 3.0, as dictated by Murray’s Law, but 2.15 and 2.38 respectively. These data will be useful in assessing patient-specific coronary physiology and tailoring revascularisation decisions.

## Introduction

First described in 1926, Murray’s law describes the physical principles for fluid flow through branched transfer networks across a wide range of biological systems. The diameters of branched vessels obey a scaling relation such that total viscous dissipation and material storage energy costs are minimised. In human arteries, Murray’s law characterises the optimal counterpoise between frictional and metabolic forces. Thus, the energy required to drive blood through the coronary circulation (overcoming vascular resistance) is balanced against the energy required to produce and maintain the blood volume such that overall energy costs are minimised (Murray, 1926). Murray’s law characterises the tapering of the arterial diameter (D) as daughter-branches (DB) arise from a parent vessel (PV) and where k is a constant, the proportionality of volumetric flow rate (Q) to diameter:

$$D_{PV}^{3}\;=\;D_{DB1}^{3}+D_{DB2}^{3}$$

and

$$Q=kD^{3}$$

A consequence of the exponent of 3.0 in Murrays’ Law, is that wall shear stress (the frictional force per unit area acting on the endothelium) is constant throughout the branching tree. Murray’s law however, assumes steady, laminar flow of a Newtonian fluid through rigid tubes of constant volume. In contrast, flow in human coronary arteries is pulsatile and may be disturbed in the context of atherosclerotic stenoses. Arteries are also distensible vessels, carrying a fluid whose mechanical properties vary with vessel radius. Theoretical derivations of turbulent flow suggest the exponent of proportionality that Murray originally proposed (3.0) may range from 2.33 to 3.0, with lower values being appropriate in more disturbed flow (Uylings, 1977). Furthermore, a range of studies, based on prototypical geometries in a wide range of practical networks, applied to Newtonian and non-Newtonian fluids largely recover the essential form of Murray’s law with varying exponent values (Bejan et al., 2000; Revellin et al., 2009; Miguel, 2019; Soni et al., 2020; Miguel, 2021). Morphometric analyses of coronary arterial trees suggest that the exponent may even lie outside theoretical limits, with reported values ranging from 2.06 to 3.20 (Hutchins et al., 1976; Zhou et al., 1999; Kassab, 2006, 2007; van der Giessen et al., 2011; Li et al., 2021).

In human coronary arteries, Murray’s law is central to all analyses of anatomy, physiology, morphometric scaling, shear-stress, wave and pressure-transmission mechanics, and predictive modelling. It is therefore relevant in clinical cardiology, even if many practitioners are unaware of it. In the cardiac catheter laboratory, the appropriate size of parent and daughter branches is deduced from a number of cues; often, pragmatically, the diameter of “normal” segments of downstream branches, but Murray’s law and Finet’s rule (Finet et al., 2008) (a derivation of Murray’s law) are also used. Decisions regarding stent sizing at bifurcation points, for example, can be made on that basis. Furthermore, the minimum diseased lumen area (MLA) triggering ischemia of a diseased left main coronary artery (≥6 mm^2^) recommended by current guidelines for lesion assessment (Neumann et al., 2019) was derived from Murray’s law (de la Torre Hernández et al., 2007; de la Torre Hernandez et al., 2011).

Recently, we developed a novel computational fluid dynamics (CFD)-based method for simulating human coronary artery physiology called virtuQ™, capable of quantifying absolute coronary blood flow (Q_CFD_) and microvascular resistance (Rmicro_CFD_) (Morris et al., 2020). In this study, we adapted the 3D CFD method to simulate side branch (SB) flow, the magnitude of which was dependent upon the exponent used in Murray’s law (Gosling et al., 2020).

The aim of this study was to apply our novel CFD-based physiological method to determine the most accurate exponent of Murray’s law in human epicardial coronary arteries and validate this against invasive clinical measurements.

## Materials and Methods

This was a retrospective analysis of clinical data collected at the Catharina Hospital, Eindhoven, NL. Computational analysis was performed at the University of Sheffield, United Kingdom. Patients provided informed consent and the study was approved by the research and ethics boards. All supporting data are provided in this manuscript or in the Supplementary Material.

### Clinical Data Collection

Adult patients undergoing clinically-indicated invasive angiography for the assessment of chest pain were included. During angiography, fractional flow reserve (FFR) and absolute coronary blood flow (Q_CIT_) and microvascular resistance (Rmicro_CIT_) were assessed in the artery of interest, with the PressureWire™ X (Abbott, MN, United States) and the continuous infusion thermodilution (CIT) method using the Rayflow™ infusion catheter (Hexacath, Paris, Fr) and the Coroventis™ (Abbott, Plymouth, MN) system (Aarnoudse et al., 2007; van ‘t Veer et al., 2016). Pseudonymised clinical imaging (DICOM) and physiological data were exported to the University of Sheffield for analysis. Cases were excluded if the physiological data were incomplete, the angiogram quality was insufficient for arterial reconstruction, or if there were major arterial SBs within 3.0 cm of the Rayflow™ infusion port. The latter exclusion is recommended by the manufacturer because significant SBs can affect the Q_CIT_ result (Aarnoudse et al., 2007). A recommended correction for the haemodynamic effect of the infusion catheter is detailed in the Supplementary Material S1. The angiographic requirements for reconstruction have been published (Ghobrial et al., 2021). Percentage stenosis was graded visually by a panel of interventional-cardiologists and additionally, with 2D- and 3D-quantitative coronary angiography (QCA).

### Simulating Coronary Blood Flow

The virtuQ method for reconstructing 3D coronary anatomy and simulating Q_CFD_ and Rmicro_CFD_ has been validated and published (Morris et al., 2020; Ghobrial et al., 2021; Solanki et al., 2021). In brief, 3D arterial anatomy of a single vessel is reconstructed from two angiographic planes, acquired ≥30° apart, using an epipolar line transection method. The relatively gentle taper of vessels means that recirculation regions are not encountered and the proximal and distal outlet manifolds to accommodate profile development are deemed unnecessary. Simulations applied standard blood parameters (density 1,056 kg/m^3^; viscosity 0.0035 Pa s). A 3D CFD simulation was then performed with the invasively measured pressures as inlet and outlet boundary conditions, which corresponded to the locations of proximal and distal pressure and temperature measurements. Simulations modelled incompressible, laminar, steady Newtonian flow in the reconstructed artery. The suitability of the steady flow approximation has previously been demonstrated in Morris’ 2015 examination (Morris, 2015) and was confirmed via analysis of Reynolds number at each model inlet, outlet and point of maximal stenosis. Furthermore, the epicardial arteries are of sufficiently large diameter to reliably model blood as a Newtonian fluid (Huckabe and Hahn, 1968). Principal model outputs were inlet and outlet Q_CFD_ and Rmicro_CFD_.

### Simulating Side Branch Flow

In this study, the virtuQ™ method was adapted to additionally simulate SB blood flow across the wall of arterial reconstructions, so that Q_inlet_ > Q_outlet_ and Q_SB_ = Q_inlet_-Q_outlet_. In a CFD analysis of a tube of varying cross section, conservation of mass dictates that the flow at every cross section is the same. If a vessel is tapered, the velocity and the wall shear stress increase as the vessel diameter reduces. A consequence of Murray’s Law is constant wall shear stress. At an arterial bifurcation the proximal flow must be shared between the two distal vessels depending on their diameters. A simple analysis protocol to compensate for branches that are clearly visible, but not included in the 3D arterial model, is to remove flow through the vessel wall at the bifurcation according to a power law representation (a generalisation of Murray’s Law) of the flow at the bifurcation. The same approach can readily be extended to smaller vessel branches that are not visible on the angiogram if it is assumed that the tapering of the primary vessel under analysis has developed, in the healthy state, according to a power law relationship between flow and diameter. Thus, it is assumed that flow must be leaking from the vessel wall to feed the myocardium through the small vessels that are not visible on the angiogram, with the remaining flow in the vessel under analysis reducing consistently as the vessel tapers. This can be handled in the CFD analysis in exactly the same way as the visible bifurcations, by prescribing a wall leak that is a function of the taper. Our model of vessel leakage therefore represented some average of the bifurcations and is intended to describe effects in real coronary arteries. The collection of unresolved SBs was therefore, characterised by a distribution of radii and geometry, ranging from the small calibre *vasa vasorum* (Gössl et al., 2003) to the largest, named arterial branches, and no single homothety relationship applies. The mathematical derivation of the porous wall method is described in the Supplementary Material S2.

Reconstructions with a total taper (D_outlet_-D_inlet_) less than one pixel (0.265 mm) were excluded because such small diameter changes cannot be accurately discerned, and so SB flow cannot be modelled reliably. Simulations were performed applying Murray’s exponents of 1.0, 1.5, 2.0, 2.5, 3.0, 3.5. The magnitude of SB flow was then plotted against the relevant exponent and an exponential function was used to fit the relationship. The optimal exponent was then interpolated from this function as the value associated with the best agreement between the CFD method and the invasive CIT measurements. All processing was performed blind to the invasive clinical results.

### Statistical Analysis

Categorical variables are presented as frequency (percentage). Continuous variables with a normal distribution are presented as mean (standard deviation) and those with a non-normal distribution as median (interquartile range). The Shapiro-Wilk test was used to assess the normality of data distribution. Between-variable correlation was assessed with Pearson’s correlation coefficient (r) and agreement with Bland Altman plots, calculating the 95% limits of agreement. The optimal value for Murray’s exponent was considered as the value with the smallest bias (mean delta) and narrowest 95% limits of agreement when comparing CFD-derived and invasive measurements of Q and Rmicro. Log transformation was used for skewed datasets.

## Results

### Case Exclusions

**FIGURE 1:**
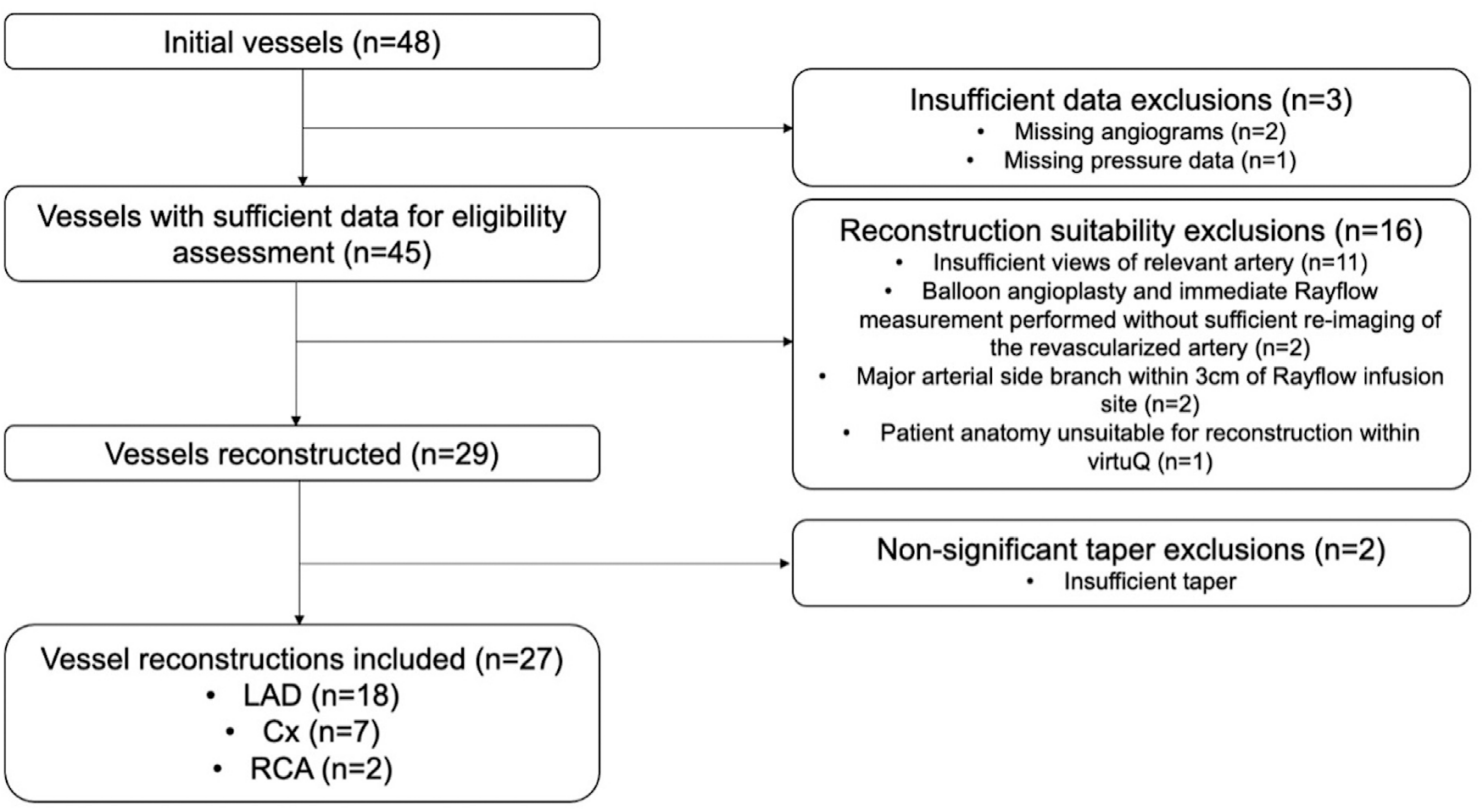
Flow diagram showing case exclusions. Cx, Left circumflex artery; LAD, Left anterior descending artery; RCA, Right coronary artery.

Forty-eight separate patient cases were assessed. Two had insufficient angiographic data and one case had incomplete pressure data. Of the remaining 45 cases, 16 were excluded due to: insufficient angiographic views (n = 11), percutaneous coronary intervention had been performed before Q_CIT_ measurement (n = 2), major arterial SBs being present within ≤3.0 cm of the CIT infusion port (n = 2), and arterial anatomy precluding reconstruction (n = 1). Twenty-nine arterial cases were successfully processed, and two of these were excluded due to insufficient arterial taper. Twenty-seven cases were therefore included in the final analysis (Figure 1).

### Patient Characteristics

Mean age was 62 (±10) years, 35% were male, mean body mass index (BMI) was 25.2 (±3.6) kg/m^2^ and four (20%) were current or ex-smokers. Full details, including demographics, comorbid conditions, and medical therapy are summarised in Table 1.

**TABLE 1:** Recruited patient characteristics.

Demographics		
Number of patients	-	20
Age (years)	-	62 ± 10
Male gender	-	7 (35%)
Body mass index (kg/m^2^)	-	25.2 ± 3.6
Current smoker	-	2 (10%)
Previous smoker	-	2 (10%)
Comorbidities		
Hypertension	-	8 (40%)
Dyslipidaemia	-	8 (40%)
Type 2 diabetes mellitus	-	1 (5%)
Previous myocardial infarction	-	4 (20%)
Previous stroke	-	1 (5%)
Left ventricular ejection fraction	Good	17 (85%)
	Moderate	1 (5%)
	Poor	0
	Unknown	2 (10%)
CCS grade^a^	0	5 (25%)
	I	10 (50%)
	II	5 (25%)
	III	0
	IV	0
NYHA grade	0	19 (95%)
	1	1 (5%)
	2	0
	3	0
	4	0
Medication		
Statin	-	15 (75%)
Aspirin	-	10 (50%)
Non-aspirin anti-platelet	-	5 (25%)
ACEi or ARB	-	8 (40%)
Anti-coagulant	-	3 (15%)
Beta-blocker	-	9 (45%)
Calcium channel-blocker	-	9 (45%)
Nitrate	-	9 (45%)
Oral hypoglycaemic agent	-	1 (5%)

Data presented as absolute number (%) or mean ± standard deviation.

a Canadian Cardiovascular Society CCS grade.

CCS, chronic coronary syndrome; NYHA, New York Heart Association functional classification of heart failure; ACEi, angiotensin-converting enzyme inhibitor; ARB, angiotensin II receptor blocker. Comorbidities and medications with a frequency of zero not presented.

### Baseline Arterial, Lesion and Physiological Characteristics

The 27 arterial cases comprised 18 left anterior descending (LAD) arteries, seven left circumflex (LCX) arteries and two right coronary arteries (RCA). Mean Q_CIT_ was 219 ml/min (±61 ml/min) and median Rmicro_CIT_ was 360 mmHg min/L (IQR 290–450 mmHg min/L). Characteristics of individual vessels reported in Supplementary Table S1.

### Optimal Murray’s Exponent Based on Absolute Coronary Blood Flow

**FIGURE 2:**
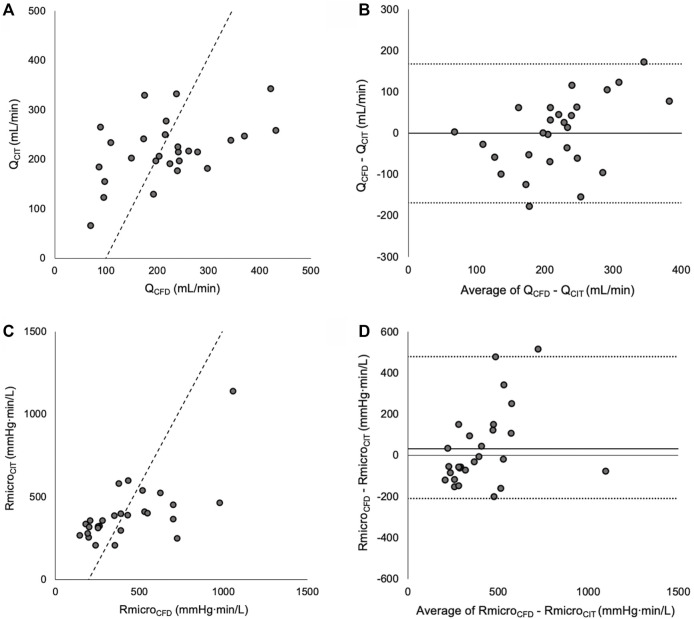
**(A)** Correlation between Q_CFD_ and Q_CIT_ (Line of best fit is Passing and Bablok). **(B)** Bland Altman plot showing mean bias and 95% limits of agreement between Q_CFD_ and Q_CIT_. **(C)** Scatter plot showing correlation between Rmicro_CFD_ and Rmicro_CIT_. **(D)** Bland Altman plot of reverse-transformed data, showing mean bias and 95% limits of agreement.

Murray’s exponent was first optimised for Q between the CIT and CFD methods. The exponent associated with the lowest mean delta, strongest correlation and best agreement (narrowest 95% limits of agreement) between Q_CFD_ and Q_CIT_ was 2.15. Differences between Q_CFD_ and Q_CIT_ were normally distributed (*p* = 0.966). When an exponent of 2.15 was applied to the CFD method, the mean delta (bias) between the methods were -168 to +168 ml/min (Figure 2) (Reconstruction measurements, flow rates and Reynolds numbers reported in Supplementary Table S2).

### Optimal Murray’s Exponent Based on Absolute Microvascular Resistance

Murray’s exponent was then optimised for Rmicro. The exponent associated with the lowest mean delta, strongest correlation and best agreement between log-transformed Rmicro_CFD_ and Rmicro_CIT_ was 2.38. Differences between log-transformed RmicroCFD and RmicroCIT were normally distributed (*p* = 0.662). When an exponent of 2.38 was applied to the CFD method, the mean delta (bias) between Rmicro_CFD_ and Rmicro_CIT_ was +30 mmHg min/L, there was a statistically significant correlation between the methods (r = 0.65, *p* < 0.001) and the 95% Bland Altman limits of agreement were -210 to +480 mmHg min/L (Figure 2).

## Discussion

In this study, we have derived the value for Murray’s exponent that best fits human epicardial coronary anatomical and physiological data. The optimum value was 2.15 based upon Q data, and 2.38 based upon Rmicro data. This is the first study to derive Murray’s exponent using a physiological approach, based upon optimised CFD analysis of absolute Q and Rmicro.

### Deviation From Theoretical Estimates

The optimum exponent of 2.15 deviates from Murray’s original law (Murray, 1926) and other analyses of geometries, homotheties and flow as related to bifurcations (Uylings, 1977; Sherman, 1981; Bejan et al., 2000; Revellin et al., 2009; Miguel, 2018; Soni et al., 2020). However, the clinical data used in this physiological-based approach captures the combined effects of numerous known and unknown factors that influence the exponent. The coronary circulation is a network of vessels that continually adapts to a multitude of factors on both the macro and microscopic level. Therefore, it is currently unclear what factor(s) contribute to this lower exponent. It is hypothesised the cyclical variation in shear forces exerted on endothelium, generated by the pulsatility of flow, may play a role, but further research is required on this area.

### Previous Estimates of Murray’s Exponent

Previous investigations of Murray’s exponent within human epicardial arteries have relied almost exclusively upon morphometric analysis of parent and daughter vessel diameters. Porcine and murine studies derived values for Murray’s exponent ranging from 2.06 to 2.72 (Zhou et al., 1999; Kassab, 2006, 2007; Li et al., 2021). However, this value may overestimate the true value in epicardial arteries, which tends to be lower than that derived from the penetrating and arteriolar branches (Zamir et al., 2015). Four studies have attempted to validate a Murray’s exponent of three in human epicardial arteries with limited success (Hahn et al., 2008; van der Waal et al., 2009; Schoenenberger et al., 2012; Chen et al., 2020). These studies suffer from large uncertainties, introduced through comparison of cubed values. Two studies have specifically aimed to quantify Murray’s exponent in human epicardial coronary arteries. The first used post-mortem angiographic imaging of 110 left main coronary artery bifurcations (Hutchins et al., 1976). Results were stratified by coronary artery stenosis grades 0–4, but the grading criteria was not reported. Exponent values for bifurcations graded 0, 1.0, 2.0, 3.0 and 4.0 were 3.2 ± 1.6, 2.8 ± 1.3, 2.6 ± 1.5 and 2.2 ± 2.1 respectively. The second and more recent study analysed Doppler ultrasound velocity and angiographic data in 18 un-diseased epicardial bifurcations, associated with an exponent of 2.27 (van der Giessen et al., 2011). The exponent derived from the most normally distributed data is considered the most reliable result. Applied to the current study, the Shapiro Wilk test as test of normality would support that the optimal exponent in human epicardial coronary arteries was 2.15. This is consistent with the findings of similar studies in the literature, derived from morphometric analysis, differing by only 0.12 compared with the most similar study (van der Giessen et al., 2011).

### Implications for Clinical Practice

**FIGURE 3:**
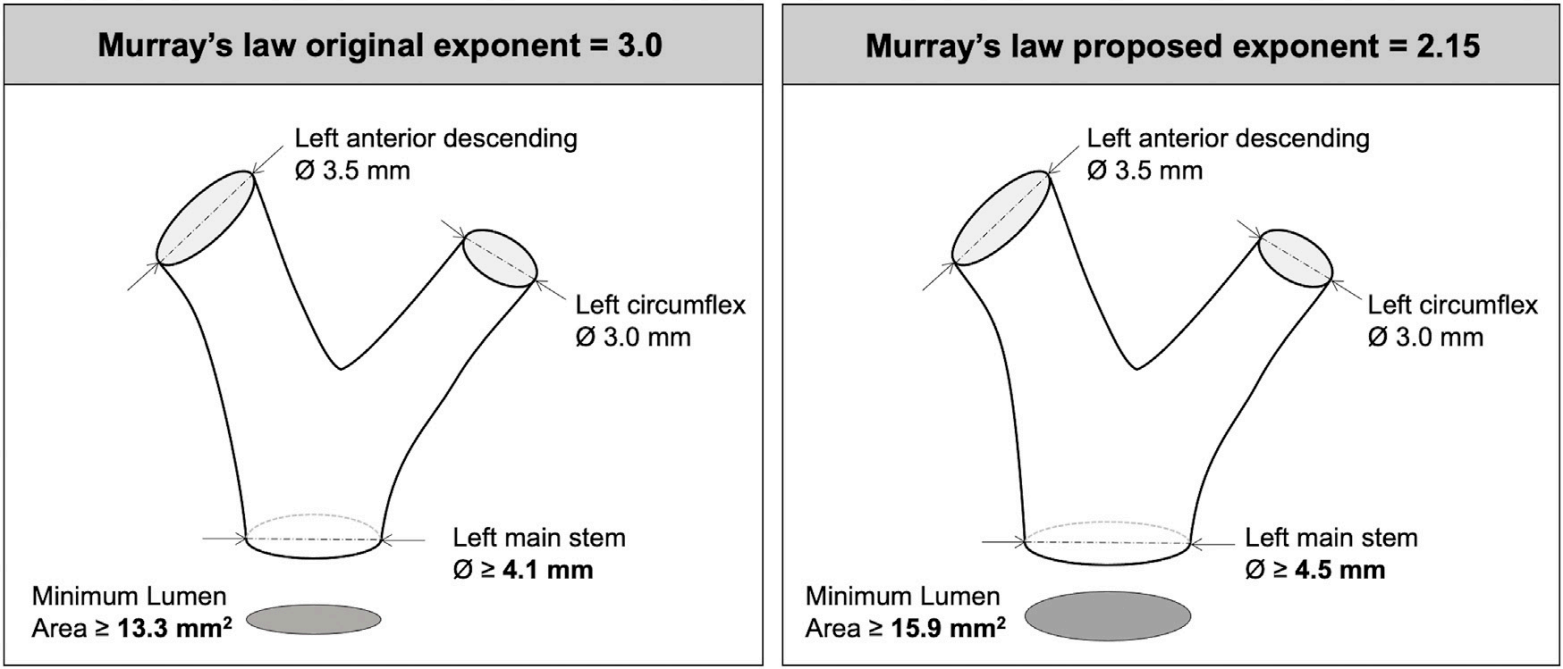
Implications of Murray’s exponent when interpreting left main bifurcation anatomy and parent daughter branch scaling.

In the context of human coronary arteries, Murray’s law (and exponent) is clinically relevant around bifurcations, with current treatment guidelines (Neumann et al., 2019) grounded in research underpinned by Murray’s law (de la Torre Hernández et al., 2007; de la Torre Hernandez et al., 2011). It is, therefore, important to understand and apply the appropriate value of Murray’s exponent for human epicardial coronary arteries. As an example, Murray’s original law (D_PV_^3^ = D_DB1_^3^ + D_DB2_^3^) dictates that, for a typical 3.5 mm downstream LAD artery and a 3.0 mm circumflex artery, the (parent) left main stem (LMS) should be 4.1 mm in diameter (MLA 13.3 mm^2^). Applying an exponent of 2.15 instead of 3.0 means that the LMS would need to be 4.5 mm in diameter (MLA 15.9 mm^2^) (Figure 3) (comment on homothety ratios detailed in Supplementary Material S3).

### Limitations

The sample size in this study was modest, but is larger than most studies in this area. More importantly, the arteries in this study were minimally diseased (INOCA cases), as assessed by FFR and percentage diameter stenosis. This has implications for the accuracy of the CFD method, which requires a pressure drop for accuracy and this may explain poorer correlation in these cases. A disproportionately high number of LAD arteries were included. The results may, therefore, not reflect the best value of Murray’s exponent in other arteries. Cases with significant SBs within 3.0 cm of the Rayflow™ infusion port were excluded as per the manufacturer’s instructions for use. These cases are likely to have had more significant taper and may have been valuable for Murray’s exponent determination. Finally, this was a retrospective analysis and so angiograms were not acquired according to the optimised protocols for 3D reconstruction; this is reflected in the percentage of case exclusions.

## Conclusion

Using a novel CFD model and invasive physiological data, we have identified the optimal exponent for Murray’s law was 2.15 for Q and 2.38 for Rmicro. This is lower than that proposed in Murray’s original law, is consistent with recent derivations based on theoretical and morphometric analyses and has clinically relevant implications.

## Data Availability

The original contributions presented in the study are included in the article/Supplementary Material, further inquiries can be directed to the corresponding author.
