# Supplementary material for: Refining Our Understanding of the Flow Through Coronary Artery Branches; Revisiting Murray’s Law in Human Epicardial Coronary Arteries
Source: Front Physiol. 2022 Apr 4;13:871912. doi: 10.3389/fphys.2022.871912 (PMC9119389; doi:10.3389/fphys.2022.871912)
Supplement: Supplementary file 1 [file DataSheet1.docx]

## Supplementary Material

**S1. Catheter correction**

Because the Rayflow™ catheter itself reduces the effective diameter of the artery, a correction must be applied to predict what Q would be without the catheter in situ (Q_thermc_). Where FFR_adenosine_ is the FFR measured under adenosine-induced maximal hyperaemia with the pressure-wire (without the Rayflow™ catheter) and FFR_saline_ is the FFR measured under saline-induced maximal hyperaemia with the Rayflow™ catheter in the coronary artery, Q_thermc_ was calculated as follows.

$$\text{Q}_{\text{thermc}}\text{ = }\frac{\text{Q}_{\text{therm}}}{\text{1 - (}\text{FFR}_{\text{adenosine }}\text{- }\text{FFR}_{\text{saline}}\text{)}}$$

**S2. Mathematical derivation of the porous wall method**

To solve the Navier-Stokes and continuity equations, it is necessary to know the fluid velocity vector at all points on the boundary of the region of space occupied by the fluid. This boundary must completely encompass the flow domain. On the luminal part of its boundary, the velocity vector has a normal component, which is determined by a sequestration flux, discussed below. The luminal boundary-tangent velocity is set to zero, consistent with the viscous, “no-slip” condition.

Regarding the velocity vector’s normal component, the observed arterial taper (reduction in diameter) is assumed to arise from flow sequestered by side-branches (SBs) which are not explicitly resolved in the reconstructed parent vessel. In the current study, SB flow was simulated by assigning a proxy, surface-normal velocity at the reconstructed luminal boundary, derived from the observed taper and Murray’s law. The method is analogous to the artery having a “porous” wall, through which fluid flows, at a rate determined by local geometry. Considering a tapered section of the reconstructed parent vessel (PV) with an inlet and outlet diameter $\text{D}_{\text{PV}}\text{ }$($\text{D}_{\text{DB1}}\text{<}\text{D}_{\text{PV}}$) where *p* denotes Murray’s exponent of proportionality. In the presence of arterial taper, we can therefore infer, from Murray’s Law ($\text{D}_{\text{PV}}^{\text{3}}\text{ = }\text{D}_{\text{DB1}}^{\text{3}}\text{+}\text{D}_{\text{DB2}}^{\text{3}}$), the existence of a non-resolved daughter vessel of radius $\text{D}_{\text{DB2}}$, where

$$\text{D}_{\text{DB2}}^{\text{p}}\text{=}\text{D}_{\text{PV}}^{\text{p}}\text{-}\text{D}_{\text{DB1}}^{\text{p}}$$

Hence, for the section in question, there is a sequestration flux

$$\text{Q}_{\text{seq}}\text{=k}\text{D}_{\text{DB2}}^{\text{p}}\text{=k(}\text{D}_{\text{PV}}^{\text{p}}\text{-}\text{D}_{\text{DB1}}^{\text{p}}\text{)}$$

This flux is assumed to be distributed uniformly over the luminal surface of the vessel section.

In practice, in the finite volume code used in this work, the sequestration flux distributes over a luminal surface defined by joined sections, each of which is very short in the streamwise direction. All computational mesh volume elements’ surface areas are known accurately. Hence, for a given length section of the luminal vessel boundary, its area, $\delta A$, can be accurately determined from a simple summation. $\text{Q}_{\text{seq}}$ above is then distributed uniformly over area $\delta A$, the resultant normal velocity, $\text{v}_{\text{n}},$ being

$$\text{v}_{\text{n}}\text{=}\frac{\text{Q}_{\text{seq}}}{\text{δA}}$$

In theory, one can model a vessel section on a conic frustum, of “height” $\text{Δl}$ and average diameter $(D_{PV}+D_{DB1})/2$. When the sequestration flux is assumed to be distributed with axial symmetry, we obtain

$v_{n}=\frac{2Q_{seq}}{\pi\left( D_{PV}+D_{DB1} \right)\Delta l}=\frac{2k(D_{PV}^{p}-D_{DB1}^{p})}{\pi\left( D_{PV}+D_{DB1} \right)\Delta l}$ (S1)

Finally, the method discussed above may be further simplified by length-averaging, or homogenising, the taper along the whole length of the vessel, then taking the rate of change of vessel diameter with distance along the vessel, $\frac{\text{dD}}{\text{dl}}$, to be small (formally, of the first order of size). One can then show for local leak velocity, equation (S1), produces the following proportionality (where *D* is local vessel diameter)

$\text{v}_{\text{n}}\text{∝}\text{D}^{\text{p-2}}$

with the constant of proportionality given by the expression $\left( \frac{4kp}{\pi}\frac{dD}{dl} \right)$.

**S3. Homothety ratio of daughter vessels detailed in figure 3**

The schematic in Figure 3 exemplifies the clinical consequences of a shift in the value of the Murray exponent in a representative left main stem arterial bifurcation. We used this example because it is the most important clinical example. But we have argued that no single bifurcation geometry is clinically characteristic. Within our model, the ratio between a major (angiographically resolved) vessel and unresolved SBs at each increment of streamwise length is effectively controlled by the observed main vessel taper and *p*. While no single homothety relationship exists, then, it is still informative to represent the heterogeneous branches in an increment of streamwise artery length, $\delta s$, by a single “fictitious” SB, to determine an effective, or average, daughter radius ratio, as follows. For a given Murray exponent, $R_{PV}^{P}=R_{DB1}^{P}+R_{DB2}^{P}$. Assume daughter DB2 (DB1) is unresolved (resolved). The taper in vessels PV and DB1 is $\frac{dR}{ds}<0$ , which is taken to be of the second order of size. Straightforward application of the binomial theorem yields

$R_{DB2}\approx p\left\| \frac{dR}{ds} \right\|\delta s$ , $R_{DB1}\approx R_{PV}+\frac{dR}{ds}\delta s$

From the above two relations, we obtain the following expression for the ratio of the radii of our fictitious daughter vessel, DB2 and the main daughter vessel PV / DB1

$\frac{R_{DB2}}{R_{DB1}}=\frac{1}{R_{PV}}p\left\| \frac{dR}{ds} \right\|\delta s+O \left( \left( \frac{dR}{ds} \right)^{2} \right)$

| ***Supplementary Table 1*** *Reconstructed vessel characteristics* | | | | | | | | | | |
| --- | --- | --- | --- | --- | --- | --- | --- | --- | --- | --- |
| **Case** | | **Hyperaemia** | | | **Rayflow results** | | | **Angiographic percentage stenosis** | | |
| **Number** | **Vessel** | **P_a_** | **P_d_** | **FFR** | **Infusion rate** | **Q_CIT_** | **Rmicro_CIT_** | **Operator** | **2D-QCA** | **3D-QCA** |
| 01 | LAD | 100 | 87 | 0.87 | 25 | 343 | 250 | 10 | 16 | 11 |
| 02 | LAD | 74 | 61 | 0.82 | 25 | 182 | 340 | 0 | 15 | 22 |
| 03 | LAD | 90 | 62 | 0.69 | 25 | 192 | 320 | 10 | 29 | 34 |
| 04 | LAD | 76 | 63 | 0.83 | 20 | 197 | 320 | 15 | 31 | 30 |
| 05 | LAD | 81 | 67 | 0.83 | 20 | 215 | 310 | 15 | 9 | 8 |
| 06 | LAD | 98 | 83 | 0.85 | 20 | 202 | 410 | 5 | 17 | 16 |
| 07 | LAD | 58 | 49 | 0.84 | 20 | 177 | 280 | 5 | 19 | 11 |
| 08 | LAD | 70 | 57 | 0.81 | 20 | 278 | 210 | 0 | 0 | 0 |
| 09 | LAD | 90 | 77 | 0.86 | 20 | 217 | 350 | 20 | 16 | 15 |
| 10 | LAD | 79 | 68 | 0.86 | 20 | 330 | 210 | 0 | 0 | 0 |
| 11 | LAD | 88 | 85 | 0.97 | 20 | 234 | 360 | 0 | 0 | 0 |
| 12 | LAD | 102 | 75 | 0.74 | 20 | 66 | 1140 | 35 | 43 | 55 |
| 13 | LAD | 77 | 64 | 0.83 | 20 | 123 | 520 | 40 | 31 | 33 |
| 14 | LAD | 91 | 86 | 0.95 | 20 | 185 | 460 | 25 | 18 | 37 |
| 15 | LAD | 82 | 76 | 0.93 | 20 | 239 | 320 | 0 | 0 | 0 |
| 16 | LAD | 107 | 87 | 0.81 | 20 | 226 | 390 | 10 | 10 | 11 |
| 17 | LAD | 77 | 70 | 0.91 | 20 | 155 | 450 | 35 | 33 | 32 |
| 18 | LAD | 88 | 82 | 0.93 | 20 | 206 | 400 | 20 | 32 | 39 |
| 19 | Cx | 100 | 98 | 0.98 | 25 | 333 | 290 | 0 | 0 | 0 |
| 20 | Cx | 93 | 88 | 0.95 | 25 | 247 | 360 | 45 | 53 | 43 |
| 21 | Cx | 146 | 128 | 0.88 | 20 | 215 | 600 | 50 | 39 | 33 |
| 22 | Cx | 96 | 75 | 0.78 | 20 | 130 | 580 | 40 | 31 | 15 |
| 23 | Cx | 74 | 69 | 0.93 | 20 | 259 | 270 | 15 | 9 | 12 |
| 24 | Cx | 110 | 106 | 0.96 | 15 | 197 | 540 | 0 | 0 | 0 |
| 25 | Cx | 69 | 66 | 0.96 | 20 | 265 | 250 | 0 | 0 | 0 |
| 26 | RCA | 101 | 97 | 0.96 | 20 | 242 | 400 | 5 | 12 | 19 |
| 27 | RCA | 100 | 97 | 0.97 | 25 | 250 | 390 | 10 | 26 | 23 |
| P_a_, proximal pressure under adenosine-induced hyperaemia (mmHg); P_d_, distal pressure under adenosine-induced hyperaemia (mmHg); Q_thermc_, absolute coronary flow measured with Rayflow catheter after correction applied (mL/min); Rmicro_thermc_, microvascular resistance measured with Rayflow catheter after correction applied (mmHg•min/L); QCA, quantitative coronary angiography; LAD, left anterior descending artery; Cx, circumflex artery; RCA, right coronary artery. All diameters reported in mm. | | | | | | | | | | |

| ***Supplementary Table 2*** *virtuQ reconstruction geometries, flow rates and Reynolds numbers* | | | | | | | | | | |
| --- | --- | --- | --- | --- | --- | --- | --- | --- | --- | --- |
| **Case** | | **Reconstruction geometries (mm)** | | | | **virtuQ flow rate (mL/min)** | | **Reynolds number** | | |
| **Number** | **Vessel** | **Inlet D** | **Outlet D** | **Maximal stenosis D** | **Tube length** | **Inlet** | **Outlet** | **Inlet** | **Outlet** | **Maximal stenosis** |
| 01 | LAD | 3.3 | 3.0 | 2.6 | 55.7 | 437 | 298 | 794.8 | 648.5 | 875.4 |
| 02 | LAD | 2.6 | 1.4 | NA | 43.3 | 334 | 84 | 779.0 | 372.9 | NA |
| 03 | LAD | 2.5 | 1.8 | 1.6 | 70 | 234 | 115 | 560.8 | 384.0 | 680.0 |
| 04 | LAD | 2.7 | 2.4 | 1.9 | 33.5 | 246 | 181 | 550.9 | 467.6 | 774.6 |
| 05 | LAD | 3.1 | 2.0 | 1.6 | 59.9 | 264 | 108 | 520.5 | 323.5 | 517.5 |
| 06 | LAD | 2.4 | 1.6 | 1.4 | 46 | 156 | 67 | 402.1 | 256.6 | 509.2 |
| 07 | LAD | 2.7 | 1.9 | 1.9 | 46.7 | 255 | 126 | 576.0 | 394.9 | 448.6 |
| 08 | LAD | 2.9 | 1.2 | NA | 70 | 238 | 36 | 503.3 | 182.9 | NA |
| 09 | LAD | 2.8 | 2.1 | 1.9 | 43.8 | 273 | 153 | 599.1 | 439.9 | 510.9 |
| 10 | LAD | 2.5 | 1.4 | 1.3 | 43.8 | 191 | 57 | 460.7 | 242.1 | 299.8 |
| 11 | LAD | 3.0 | 1.4 | NA | 40.9 | 121 | 23 | 245.8 | 100.6 | NA |
| 12 | LAD | 2.1 | 1.2 | 0.9 | 52.7 | 71 | 21 | 203.7 | 107.5 | 455.3 |
| 13 | LAD | 2.5 | 1.4 | 1.1 | 56.6 | 102 | 31 | 246.4 | 130.1 | 263.2 |
| 14 | LAD | 2.8 | 2.5 | 1.6 | 46.3 | 88 | 65 | 187.2 | 159.5 | 309.6 |
| 15 | LAD | 3.4 | 2.2 | NA | 47.6 | 378 | 149 | 670.2 | 407.7 | NA |
| 16 | LAD | 2.7 | 2.4 | 1.8 | 53.3 | 247 | 178 | 547.3 | 458.7 | 651.8 |
| 17 | LAD | 2.6 | 2.0 | 0.8 | 44.6 | 100 | 60 | 237.0 | 180.3 | 600.7 |
| 18 | LAD | 3.7 | 2.6 | 2.0 | 58.2 | 210 | 97 | 346.3 | 228.9 | NA |
| 19 | Cx | 4.2 | 3.0 | 2.9 | 44.4 | 252 | 124 | 362.4 | 248.3 | 374.7 |
| 20 | Cx | 4.1 | 1.9 | 1.7 | 63.4 | 423 | 81 | 627.6 | 259.9 | 431.8 |
| 21 | Cx | 3.0 | 2.2 | 1.5 | 59.1 | 294 | 159 | 603.9 | 435.5 | 725.5 |
| 22 | Cx | 2.5 | 2.0 | 1.8 | 54 | 199 | 121 | 483.6 | 371.6 | 579.8 |
| 23 | Cx | 4.0 | 2.4 | NA | 51.4 | 473 | 158 | 718.3 | 400.3 | NA |
| 24 | Cx | 3.2 | 2.7 | 2.4 | 37.4 | 204 | 133 | 381.6 | 304.1 | 388.3 |
| 25 | Cx | 2.7 | 2.4 | 1.9 | 37.2 | 91 | 71 | 206.5 | 180.9 | 242.5 |
| 26 | RCA | 3.5 | 3.1 | 2.4 | 55.9 | 176 | 140 | 310.0 | 274.1 | 416.9 |
| 27 | RCA | 3.9 | 2.9 | 2.5 | 68.2 | 225 | 123 | 352.7 | 255.1 | 469.3 |
| Diameter of maximal stenosis reported as NA for arteries without any detectable stenosis on the virtuQ reconstruction  All reported flow rates and Reynolds numbers processed from an exponent of 2.15  Inlet D, Inlet diameter; Maximal stenosis D, Maximal stenosis diameter; Outlet D, Outlet diameter; Cx, Left circumflex artery; LAD, Left anterior descending artery; RCA, Right coronary artery | | | | | | | | | | |
